# An Electrosurgical Endoknife with a Water-Jet Function (Flushknife) Proves Its Merits in Colorectal Endoscopic Submucosal Dissection Especially for the Cases Which Should Be Removed *En Bloc*

**DOI:** 10.1155/2013/530123

**Published:** 2013-09-23

**Authors:** Yoji Takeuchi, Toshio Shimokawa, Ryu Ishihara, Hiroyasu Iishi, Noboru Hanaoka, Koji Higashino, Noriya Uedo

**Affiliations:** ^1^Department of Gastrointestinal Oncology, Osaka Medical Center for Cancer and Cardiovascular Diseases, 1-3-3 Nakamichi, Higashinari-ku, Osaka 537-8511, Japan; ^2^Graduate School of Medicine and Engineering, University of Yamanashi, 4-3-11 Takeda, Kofu City, Yamanashi 400-8511, Japan

## Abstract

*Background*. Previously, we reported that the Flushknife (electrosurgical endoknife with a water-jet function) could reduce the operation time of colorectal endoscopic submucosal dissection (ESD) however, suitable situation for the Flushknife was obscure. This subgroup analysis of a prospective randomized controlled trial was aimed to investigate the suitable situation for the Flushknife. *Methods*. A total of 48 superficial colorectal neoplasms that underwent ESD using either the Flexknife or the Flushknife in a referral center were enrolled. The differences of operation time between the Flexknife and the Flushknife groups in each subgroup (tumor size, location, and macroscopic type) were analyzed. *Results*. Median (95% CI) operation time calculated using survival curves was significantly shorter in the Flushknife group than in the Flexknife group (55.5 min [41, 78] versus 74.0 [57, 90] min; *P* = 0.039, Hazard Ratio HR: 0.53; 95% CI (0.29–0.97)). In particular, the HR in patients with laterally spreading tumors-nongranular type (LST-NG) in the Flushknife group was significantly smaller than in the Flexknife group (HR: 0.165→0.17; 95% CI (0.04–0.66)). There was a trend of decreasing HRs according to larger lesion size. *Conclusions*. The Flushknife proved its merits in colorectal ESD especially for the lesions which should be removed *en bloc* (LST-NG and large lesion).

## 1. Introduction

Endoscopic submucosal dissection (ESD) is one of the standard treatments for large upper gastrointestinal superficial neoplasms in Japan and South Korea [1–5] because of its high *en bloc* resection (entire tumor resection in one piece) rate. ESD is also considered as a promising procedure in Western countries [6]. Although there are some reports on ESD for colorectal superficial neoplasms [7–9], ESD has not been a standard therapy for large colorectal superficial neoplasms, even in Japan. The reason is that it is time consuming, technically difficult, and related to a higher incidence of complications (perforation and bleeding) than conventional endoscopic mucosal resection (EMR) [10]. 

**Figure 1:**
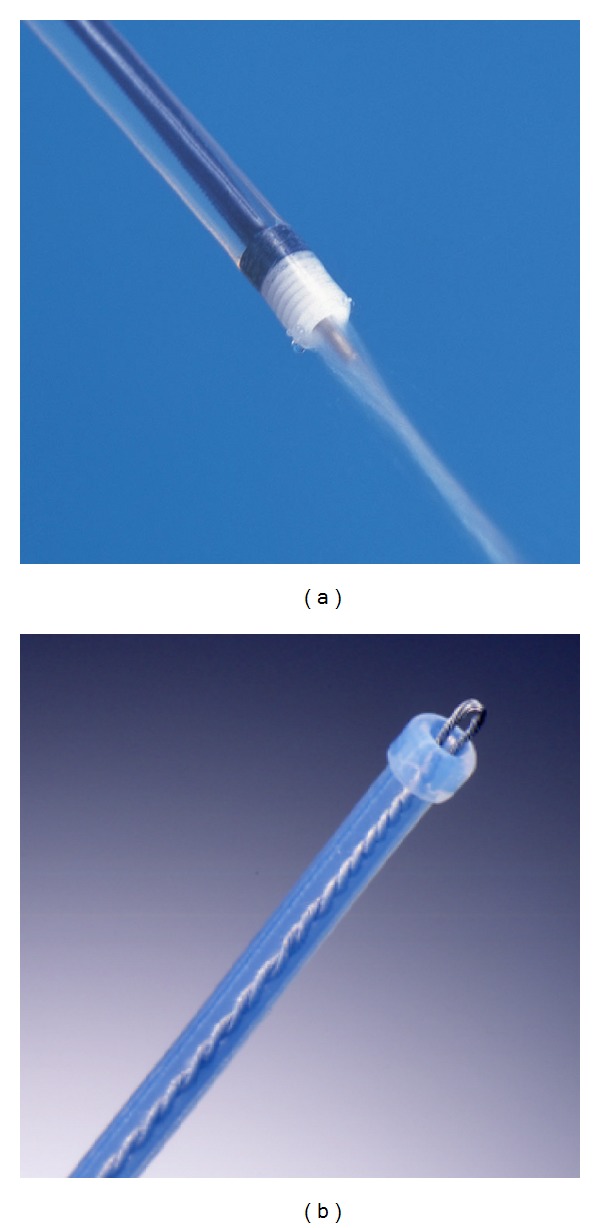
Electrosurgical endoknives used in our study. (a) The Flexknife is a flexible tool that enables endoscopists to adjust the projection length of the tip when needed and is not combined with a water-jet function. (b) The Flushknife is a short needle electrosurgical endo-knife combined with a water-jet function.

Flushknife (DK2618JN15, Fujifilm Medical, Tokyo, Japan) is a short needle electrosurgical endo-knife combined with a water-jet function (Figure 1(a)) [11]. We have reported that the Flushknife could reduce the operation time of colorectal ESD for patients with large colorectal superficial neoplasms [12], compared with a standard electrosurgical endo-knife (Flexknife; KD-630L, Olympus Medical Systems, Tokyo, Japan, Figure 1(b)) [13, 14]. In the study, we enrolled lesions larger than 20 mm and smaller than 60 mm and analyzed the data irrespective of tumor size, location of the tumor, and macroscopic type (e.g., protruded/laterally spreading tumor-granular type (LST-G) or laterally spreading tumor-nongranular type (LST-NG)/lesion with fold convergence) [15]. Therefore, the characteristics of a favorable situation for the Flushknife were obscure. In this subgroup analysis, we examined the favorable situation for the Flushknife in colorectal ESD.

## 2. Patients/Methods

The original study of this secondary analysis was designed as a randomized controlled trial following CONSORT statement performed at an endoscopy unit in Osaka Medical Center for Cancer and Cardiovascular Diseases, Japan. The original study protocol was approved by the institutional review board of Osaka Medical Center for Cancer and Cardiovascular Diseases and described in the previous report [12]. The study was registered in the University Hospital Medical Network Clinical Trials Registry (UMIN-CTR; http://www.umin.ac.jp/ctr/index-j.htm) as number UMIN000001302.

**Figure 2:**
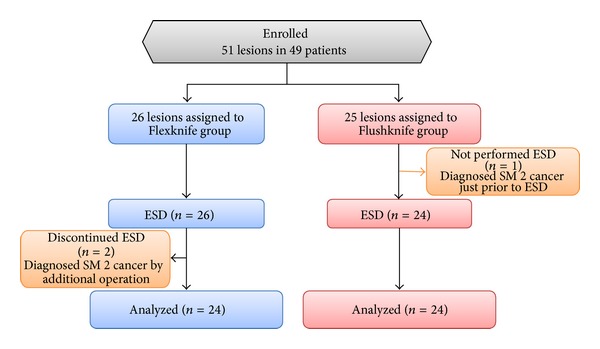
Flow diagram of the participants after enrollment. Removal of the lesions was incomplete for two cases in the Flexknife group, and one lesion removal procedure was not performed in the Flushknife group. Thus, 24 lesions in each group were completed and analyzed in this study.

Between April 2008 and March 2009, a total of 49 patients with 51 colorectal intramucosal or minutes-invaded (SM < 1000 *μ*m: SM1) neoplasms larger than 20 mm and smaller than 60 mm in diameter were enrolled in the original trial. Patients were excluded if they had noncorrectable coagulopathy, if they had severe organ failure, or if they had undergone anticoagulant therapy. Written informed consent was obtained from all patients. Twenty-six lesions were randomly assigned to the Flexknife group, and 25 lesions were assigned to the Flushknife group. After allocation, two lesions in the Flexknife group and one lesion in the Flushknife group were regarded as dropout cases, which included one lesion in the Flexknife group, in which the procedure could not be completed even using the Hookknife and finally it was diagnosed as deeply invaded submucosal cancer. Although, in the Flexknife group, the endoknife was switched to the Flushknife because of severe submucosal fibrosis in one case, the lesion was analyzed as the lesions in the Flexknife group. On the other hand, all procedures were completed using only the Flushknife in the Flushknife group. Therefore, twenty-four lesions in the Flexknife group and 24 in the Flushknife group completed ESD and data were analyzed (Figure 2). Patient characteristics and clinicopathological features for each treatment group are shown in Table 1. The following traits were not significantly different: age, sex, tumor location, tumor type, estimated size of tumor before ESD, endoscopist used, resected tumor size, resected specimen size, histological diagnosis, and resectability.

In this subgroup analysis, we reanalyzed the main outcome measurement (the difference in operation time between the groups) using survival curves at first, and then, we compared the difference in operation time between the Flexknife group and the Flushknife group in each subgroup tumor size, location, and macroscopic type. 

## 3. Statistical Analysis

We defined the operation time (measured from the start of the submucosal injection until tumor removal) as a surrogate marker for assessment of the technical difficulty of the procedure in our secondary analysis. All data were collected in our hospital and analyzed by the data center at University of Yamanashi, Japan. For the reanalysis of the main outcome measurement (the difference in operation time between the groups), we used the empirical distribution function plot to calculate survival curves and the logrank test to investigate treatment comparisons. We used the Cox proportional hazards model for the subgroup analysis. All hazard ratios (HRs) and 95% confidence intervals (CIs) in the subgroup analysis were determined by multiple individual Cox models. Interaction terms were included in the models with significance testing by the likelihood ratio test. Data analysis was conducted using the statistical package *R* 2.8.1 (http://www.r-project.org/). All *P* values were two-tailed, and *P* < 0.05 was defined as statistically significant.

## 4. Results

**Figure 3:**
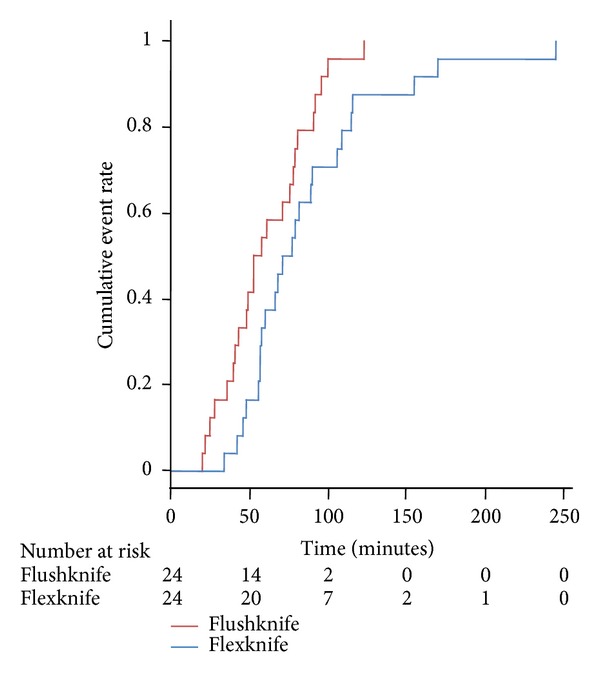
Empirical distribution function plot for operation time using each endo-knife. The hazard ratio for completion of the procedure between two groups was 0.53 (95% CI: 0.29–0.97) and was significant (*P* = 0.04).

Median (95% CI) operation time calculated using survival curves was significantly shorter for patients assigned to the Flushknife group than that for those assigned to the Flexknife group (55.5 min [41, 78] versus 74.0 [57, 90] min; *P* = 0.04, HR: 0.53; 95% CI (0.29–0.97) Figure 3). Main outcome measurement of this trial, the difference of operation time between two groups in overall patients was also approved using survival curves. 

Then, we divided the patients into subgroups according to estimated tumor size, location of the tumor, and macroscopic type. Subset for estimated tumor size consisted of three groups (<30 mm, 30–39 mm, >40 mm). 25 lesions (9 in the Flushknife group and 14 in the Flexknife group) were smaller than 30 mm. 14 lesions (9 in the Flushknife group and 5 in the Flexknife group) were larger than 30 mm and smaller than 39 mm. 11 lesions (6 in the Flushknife group and 5 in the Flexknife group) were larger than 40 mm. Subset for location of the tumor consisted of two groups (rectum, colon). 13 lesions (7 in the Flushknife group and 6 in the Flexknife group) were located in rectum. 35 lesions (18 in the Flushknife group and 17 in the Flexknife group) were located in the colon. Subset for macroscopic type also consisted of two groups (protruded and LST-G, LST-NG and lesions with fold convergence). Thirty eight lesions (19 in each group) were protruded or of LST-G type. Ten lesions (5 in each group) were LST-NG or lesions with fold convergence.

**Figure 4:**
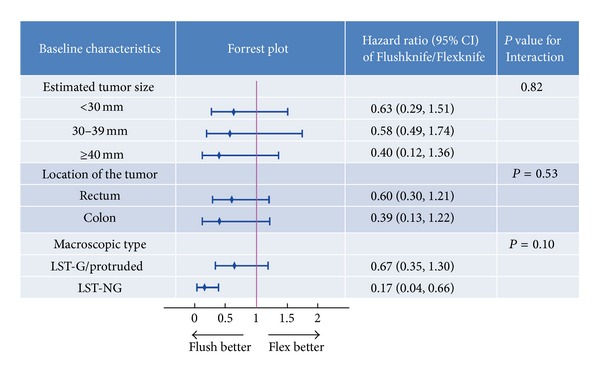
Hazard ratios for completion of the procedure and 95% CIs in each subgroup.

Almost all the conditions indicated that the Flushknife was more favorable than the Flexknife (Figure 4). In particular, HR in patients with LST-NG using the Flushknife was significantly smaller than in those using the Flexknife, suggesting that the Flushknife was significantly favorable than the Flexknife for colorectal ESD in patients with LST-NG (HR: 0.17; 95% CI (0.04–0.66)). Interaction of treatment group with these factors was not significant (*P* = 0.07). Furthermore, there was a trend of decreasing HRs for larger lesions (HR [95% CI] for <30 mm group, 0.63 [0.29–1.51]; 30–39 mm group, 0.58 [0.49–1.74]; and the ≥40 mm group, 0.40 [0.12–1.36]), suggesting that the Flushknife was more favorable than the Flexknife for larger lesion.

## 5. Discussion

In this subgroup analysis of a randomized controlled trial for colorectal ESD, we found that the Flushknife was a more favorable endo-knife than the Flexknife in patients with LST-NG and larger lesions. The efficacy of the Flushknife is already discussed in the original article that benefit of the Flushknife's water-jet function might principally result from direct injection of saline into the submucosa. Endoscopists using the Flushknife did not need to switch their devices so often—this helped to reduce the total operation time and enabled endoscopists to perform their procedure seamlessly without interruption. 

In this analysis, we defined the operation time as a surrogate marker for assessment of technical difficulty of the procedure, as the operation time is longer during a careful procedure avoiding perforation and incomplete resection. Isomoto et al. [16] examined the clinicopathological factors associated with clinical outcomes of colorectal ESD and reported that colorectal tumors with the finding of fibrosis or larger than 30 mm in diameter presented a significant risk for perforation. The technical difficulty of colorectal ESD was evaluated by incidence of incomplete resection and perforation in their article. In our study, perforation and incomplete resection occurred in only one case in each group (2%). Due to the low incidence of perforation and piecemeal resection, we were unable to assess the technical difficulty of colorectal ESD using such surrogate markers. Thus, assessing the operation time as a surrogate marker for technical difficulty, we found that the Flushknife was more favorable for treatment of LST-NG and for larger lesions. LST-NG commonly has fibrosis in their submucosa and is difficult to resect by conventional EMR or even by ESD [17]. The results suggest that the Flushknife is also more favorable for lesions that have such higher risk of complications. 

Uraoka et al. [17] reported that LST-NG had a significantly higher rate of submucosal invasion than LST-G. As such, the authors concluded that LST-NG tumors should be removed *en bloc*. Fujishiro et al. [14] reported that LST-NG resected by ESD has more frequent submucosal invasion than LST-G (44% of treated LST-NG pseudo depressed type were submucosal cancer), and suggested that this was a sufficient reason for use of colorectal ESD for LST-NG to avoid leaving cancer cells in the submucosa, and also for precise histopathological diagnosis for lymphatic vascular involvement. Tanaka et al. [18] also proposed LST-NG as an indication of colorectal ESD. Overall, these data suggest that LST-NG should be removed *en bloc* by ESD and our data suggest that they should be treated using the Flushknife. The reason that the Flushknife was more useful for especially LST-NG is unclear, but we suppose that LST-NG is known as the lesion often accompanied with fibrosis in their submucosa and it is generally known that enough submucosal space by injecting hyaluronate sodium in such lesions accompanied with fibrosis in their submucosa is easy to collapse and cannot be kept for a while. Then, the colonoscopists using the Flushknife have to repeat submucosal injection using endoscopic needle and it needs more time for changing the device. The Flushknife can inject saline into the submucosal layer directory and it also can dissect the submucosal layer just after the injection, before collapse of the submucosal cushion. Therefore, the Flushknife may be able to show the efficacy especially in LST-NG.

The *en bloc* resection rate by conventional EMR is quite low for large gastrointestinal neoplasms [19]. ESD has a high *en bloc* resection rate even for large lesions, allowing more precise histological diagnosis than conventional EMR. Actually, piecemeal resection by conventional EMR is generally considered the treatment of choice given the low chance that these lesions harbor malignancy [20, 21]. The patient must undergo repetitive surveillance colonoscopies to confirm the curability of the lesion after piecemeal resection, and an additional EMR/ESD is required if the residue of neoplasm was confirmed. On the other hand, no recurrence should theoretically be seen after *en bloc* R0 resection, and endoscopists should only be concerned about metachronous neoplasms. Thus, we suggest that the larger lesions should also be removed *en bloc* by colorectal ESD, and our data suggest that they should be treated using the Flushknife. 

A potential limitation of our study is that it is a retrospective exploratory subgroup analysis in a prospective randomized study involving only 48 subjects. Therefore, the confidence interval is wide even if the HRs were significant. Thus, our results should be interpreted with caution. To further validate our results, a new randomized controlled trial comparing the two endo-knives in patients with LST-NG or larger lesions is required. Nevertheless, the advantages of the Flushknife are generally well established, and alternative improvements for standardization of colorectal ESD should be examined in the future, such as improving hemostasis, decreasing complications, and optimizing sedation. 

In conclusion, Flushknife is more favorable than Flexknife for colorectal ESD especially in patients with LST-NG and larger lesions, which should be removed by ESD.

## Figures and Tables

**Table 1:** Baseline and clinicopathological features of the treatment groups.

	Flexknife group, *n* (%)	Flushknife group, *n* (%)
Number of patients	26	23
Number of lesions	26	25
Excluded lesions after allocation	2	1
Number of analyzed lesions	24	24
Median age (range, years old)	68 (51–86)	68 (47–87)
Sex		
Men	16 (67)	9 (38)
Women	8 (33)	15 (62)
Location of the lesions		
Rectum	7 (29)	6 (25)
Colon	17 (71)	18 (75)
Tumor type		
Protruded or LST-G	19 (79)	19 (79)
LST-NG or lesions with fold convergence	5 (21)	5 (21)
Median estimated tumor size (range, mm)	27.5 (20–50)	30 (20–60)
Endoscopist		
Y.T.	17 (73)	15 (64)
N.U.	7 (27)	9 (36)
Median resected tumor size (range, mm)	38 (12–60)	35.5 (20–95)
Median resected specimen size (range, mm)	40 (15–60)	37.5 (22–100)
Histological diagnosis		
Low-grade adenoma	7	7
High-grade adenoma	9	7
Noninvasive carcinoma	5	9
SM 1 (≤1000 *μ*m)	2	0
SM 2 (>1000 *μ*m)	1	1
Resectability		
R0	20 (83%)	19 (79%)
R1	4 (17%)	5 (21%)
Rx	0	0
